# Integrin alpha V (ITGAV) expression in esophageal adenocarcinoma is associated with shortened overall-survival

**DOI:** 10.1038/s41598-020-75085-7

**Published:** 2020-10-27

**Authors:** Heike Loeser, Matthias Scholz, Hans Fuchs, Ahlem Essakly, Alexander Iannos Damanakis, Thomas Zander, Reinhard Büttner, Wolfgang Schröder, Christiane Bruns, Alexander Quaas, Florian Gebauer

**Affiliations:** 1 Department of General, Visceral, Cancer and Transplantation Surgery, University of Cologne, Kerpener Strasse 62, 50937 Cologne, Germany; 2 Institute of Pathology, University of Cologne, Cologne, Germany; 3 Department I of Internal Medicine, Center for Integrated Oncology Aachen Bonn Cologne Duesseldorf, Cologne, Germany; 4 Gastrointestinal Cancer Group Cologne GCGC, University of Cologne, Cologne, Germany

**Keywords:** Oesophageal cancer, Surgical oncology, Tumour biomarkers

## Abstract

Valid biomarkers for a better prognostic prediction of the clinical course in esophageal adenocarcinoma (EAC) are still not implemented. Integrin alpha V (ITGAV), a transmembrane glycoprotein responsible for cell-to-matrix binding has been found to enhance tumor progression in several tumor entities. The expression pattern and biological role of ITGAV expression in esophageal adenocarcinoma (EAC) has not been analyzed so far. Aim of the study is to evaluate the expression level of ITGAV in a very large collective of EAC and its impact on individual patients´ prognosis. 585 patients with esophageal adenocarcinoma were analyzed immunohistochemically for ITGAV. The data was correlated with clinical, pathological and molecular data (TP53, HER2/neu, c-myc, GATA6, PIK3CA and KRAS). A total of 85 patients (14.3%) out of 585 analyzable tumors showed an ITGAV expression and intratumoral heterogeneity was low. ITGAV expression was correlated with a shortened overall-survival in the patients´ group that underwent primary surgery (p = 0.014) but not in the group of patients that received neoadjuvant treatment before surgery. No correlation between any of the analyzed molecular marker (mutations or amplifications) (TP53, HER2, c-myc, GATA6, PIK3CA and KRAS) and ITGAV expression could be observed. A multivariate cox-regression model was performed which showed tumor stage, lymph node metastasis and ITGAV expression as independent prognostic markers for overall-survival in the group of patients without neoadjuvant treatment. ITGAV expression is correlated with an impaired patient outcome in the group of patients without neoadjuvant therapy and serves as a prognostic factor in EAC.

## Introduction

Esophageal cancer is the eighth most common cancer and sixth leading cause of cancer death in the world^1^. While advantages in perioperative treatment have been achieved including establishment of standardized perioperative treatment protocols and surgical procedures with acceptable low perioperative morbidity and mortality rates, the overall-survival of patients with esophageal adenocarcinoma (EAC) remains limited^2^. Today, treatment response prediction or even stratification into high- and low-risk tumors is hardly possible or only done by clinical parameter. Therefore, the identification of individual prognostic markers is of high importance, as there is a high fraction of patients receiving multimodal treatment with only limited or even without any histopathological response and therefore limited individual benefit considering long-term survival^3^.

Integrins are a family of cell-adhesion molecules that consist of two linked heterodimeric subunits, the α and β subunit and mediate cell–cell and cell-to-extracellular matrix (ECM) adhesions^4^. The combination of the particular α and β subunit determines the receptor specificity, however, integrins are physiologically involved in promoting signaling pathways that regulate proliferation, cell survival, and migration^5^. Integrins have become of interest in cancer research as there are distinct roles of certain Integrin α/β combinations in carcinogenesis and tumor progression, with focus on metastatic processes and interactions between tumor cells and the ECM^6^. Integrin αV (ITGAV) consists of five members αvβ1, αvβ3, αvβ5, αvβ6 and αvβ8 and is part of the receptors for fibronectin, vitronectin, fibrinogen. It was previously found overexpressed in different tumor types and partly associated with shortened overall-survival (OS)^7–10^. However, the expression pattern and biological role of ITGAV expression in esophageal adenocarcinoma (EAC) has not been analysed so far. Furthermore, as integrins are important in cancer progression, a systematic analysis of ITGAV expression and known molecular alterations in EAC was performed. The aim of the present study is therefore to analyze the expression of ITGAV in a collective of almost 700 patients with EAC and correlate the expression profile to clinico-pathological, molecular (TP53, Her2/neu, c-myc, GATA6, PIK3CA and KRAS amplification) and survival data.

## Patients and methods

### Patients and tumor samples

Formalin-fixed and paraffin embedded tumor tissue of 685 patients with esophageal adenocarcinomas that underwent primary surgical resection or resection after neoadjuvant therapy at the Department of General, Visceral and Cancer Surgery, University of Cologne, Germany was analyzed as previously described^11,12^. The standard surgical procedure consisted of a transthoracic en-bloc esophagectomy with two-field lymphadenectomy (abdominal and mediastinal lymph nodes), reconstruction by formation of a gastric tube with intrathoracic esophagogastrostomy (Ivor-Lewis esophagectomy)^13^. The abdominal phase was predominantly performed as a laparoscopic procedure (hybrid Ivor-Lewis esophagectomy). Technical details of this operation are described elsewhere^14–16^. Patients with locally advanced esophageal cancer (cT3) or evidence for loco regional lymph node metastasis in clinical staging received preoperative chemoradiation (5-Fluouracil, cisplatin, 40 Gy) or chemotherapy alone. Follow-up data were available for all patients (Table 1).

**Table 1:** Clinico-pathological data of the entire patients cohort and cross-table analysis of Integrin alphaV expression.

	Total	Integrin αV expression		
		Negative	Positive	p value
**Sex**				
Female				
No	75	61	14	
%	12.6%	81.3%	18.7%	
Male				
No	520	449	71	
%	87.4%	86.3%	13.7%	0.288
**Age group**				
< 65 years				
No	309	256	53	
%	51.9%	83.0%	17.0%	
> 65 years				
No	286	254	32	
%	48.1%	88.6%	11.4%	0.056
**Tumor stage**				
pT1				
No	86	75	11	
%	14.5%	87.2%	12.8%	
pT2				
No	74	68	6	
%	12.5%	91.9%	8.1%	
pT3				
No	411	351	60	
%	69.4%	85.4%	14.6%	
pT4				
No	19	11	8	
%	3.2%	57.9%	42.1%	0.005
**Lymph node metastasis**				
pN0				
No	238	210	28	
%	40.3%	88.2%	11.8%	
pN +				
No	352	296	56	
%	59.7%	84.1%	15.9%	0.187
**UICC**				
I				
No	121	106	15	
%	20.6%	87.6%	12.4%	
II				
No	142	127	15	
%	24.1%	89.4%	10.6%	
III				
No	251	207	44	
%	42.7%	82.5%	17.5%	
IV				
No	74	64	10	
%	12.3%	86.5%	13.5%	0.245
**Neoadjuvant treatment**				
No				
No	262	228	34	
%	44.0%	87.0%	13.0%	
Yes				
No	333	282	51	
%	56.0%	84.7%	15.3%	0.479

Single spot tissue micro arrays (TMA) were built for immunohistochemical analyses. TMA construction was performed as previously described^12,17^. In brief, tissue cylinders with a diameter of 1.2 mm each were punched from selected tumor tissue blocks using a self-constructed semi-automated precision instrument and embedded in empty recipient paraffin blocks. For the multi-spot TMA (165 patients), up to eight tumor spots were punched out of the tumour, four spots each from the surface and the invasion front. The multi-spot array should answer the question of heterogeneity of an ITGAV expression within the tumor. Four μm sections of the resulting TMA blocks were transferred to an adhesive coated slide system (Instrumedics Inc., Hackensack, NJ) for immunohistochemistry. All procedures performed in studies involving human participants were in accordance with the ethical standards of the institutional research committee and with the 1964 Helsinki declaration and its later amendments or comparable ethical standards. The present study was ethically approved by the University of Cologne Ethics Committee (reference no. 13-091) and written informed consent was obtained from all patients.

### Immunohistochemistry for Integrin alpha V (ITGAV)

Immunohistochemistry (IHC) was performed on TMA slides using the Integrin alpha V rabbit monoclonal antibody (ab150361; dilution 1:300; Abcam, UK). Staining and scoring procedures were conducted as previously described^12,18–20^. All immunohistochemical stainings were performed using the Leica BOND-MAX stainer (Leica Biosystems, Germany) according to the protocol of the manufacturer.

The membraneous staining pattern was scored manually and independently by two pathologists (A.Q. and H.L.) according to a 4-tier-scoring system. Score 3 + was defined as a strong staining of ≥ 30% of tumor cells or moderate staining ≥ 70%. A weak staining in > 70% or moderate staining in > 30 and ≤ 70% or as strong staining in ≤ 30% of tumor cells was considered as Score 2 + . Score 1 + was assigned when ≤ 70% of tumor cells were weakly positive or ≤ 30% were moderately stained. Less staining was defined as negative (Score 0). Discrepant results were resolved by consensus review.

Expression of Integrin alpha V was correlated with molecular markers including analysis of TP53, Her2/neu, c-myc, GATA6, PIK3CA- and KRAS amplification. A detailed description of the analysis of TP53, KRAS, PIK3CA, Her2/neu and GATA6 is already published^11,12,19,21^.

### Statistical analysis

Clinical data were collected prospectively and analyzed according to a standardized protocol as previously described^11,12^. SPSS Statistics for Mac (Version 21, SPSS) was used for statistical analysis. Interdependence between stainings and clinical data were calculated using the chi-squared and Fisher’s exact tests, and displayed by cross-tables. Survival curves were plotted using the Kaplan–Meier method and analyzed using the log-rank test. All tests were two-sided. P values < 0.05 were considered statistically significant.

## Results

### Patients’ baseline characteristics

On the TMA a total of 585 patients of 685 (86.7%) were immunohistochemically interpretable for ITGAV. Reasons for the non-informative cases were missing tissue samples or the absence of distinct cancer tissue in the TMA spot. Clinicopathological data is depicted in Table 1. Patients were predominantly men (male n = 520, 87.4%, female n = 75, 12.6%). The median age of the entire patient cohort at the time of diagnosis was 65.2 years (range 33.6–85.6 years). In 333 patients (56.0%) a neoadjuvant treatment (chemo- or radiochemotherapy) was performed before surgery.

### Expression of ITGAV in esophageal adenocarcinoma

**Figure 1:**
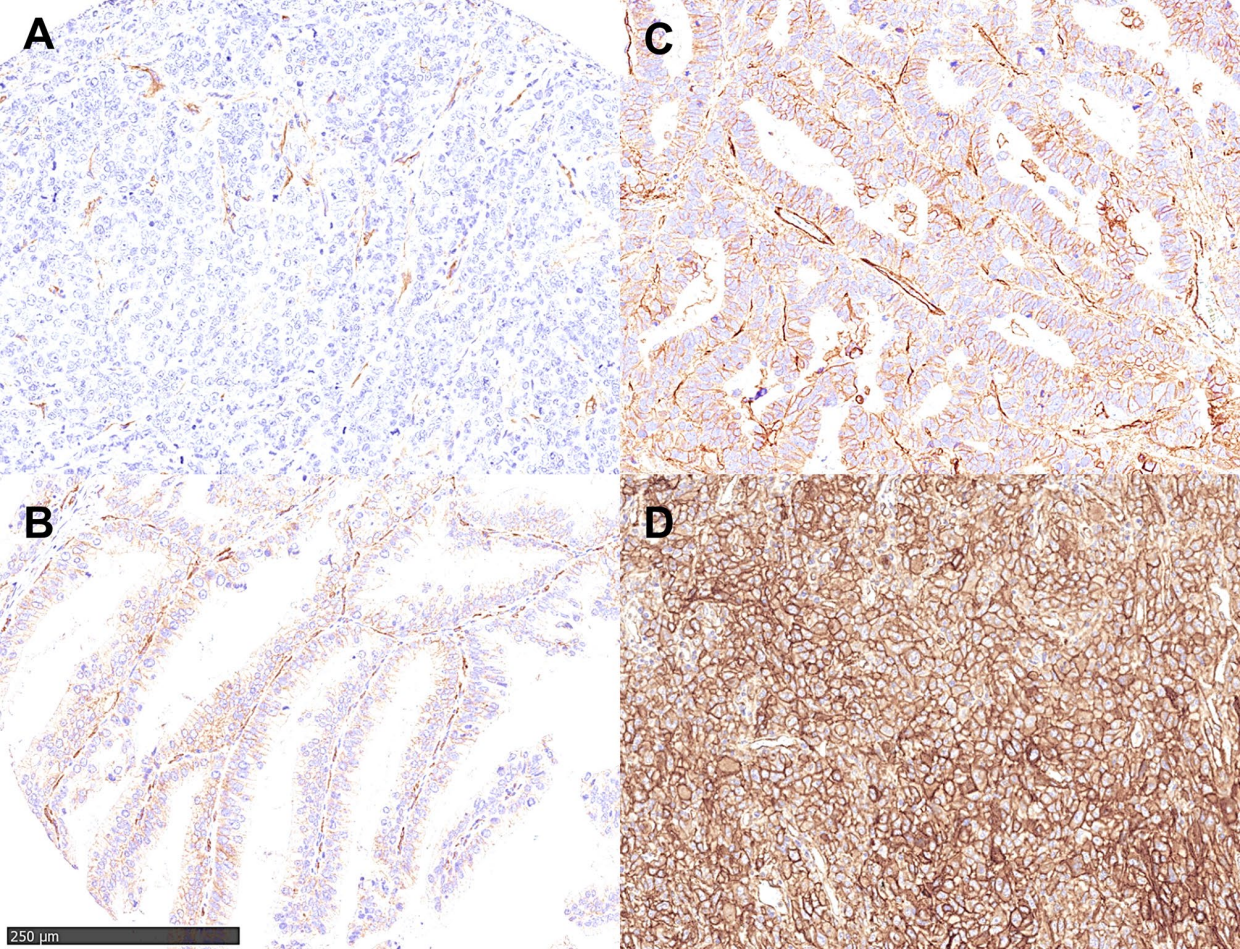
Immunohistochemistry of ITGAV in EAC: negative staining (Score 0) with internal positive control in (**A**), weak membranous staining (Score 1 +) in (**B**), moderate staining (Score 2 +) in (**C**), strong staining in all tumor cells (Score 3 +) in (**D**).

Expression of ITGAV was detectable in 85 patients (14.3%) (Fig. 1). In cross table analysis a correlation between ITGAV expression, older patients (> 65 years) and advanced tumor staged could be revealed (p = 0.05 and p = 0.005, respectively). No correlation between any of the analyzed molecular marker (TP53 mutation, HER2/neu, c-myc, GATA6, PIK3CA and KRAS amplifications) and ITGAV expression was seen (Table 2).

**Table 2:** Correlation between molecular data and ITGAV expression.

	Total	Integrin αV expression		
		Negative	Negative	Negative
**TP53**				
Negative				
No	149	131	18	
%	42.3%	87.9%	12.1%	
Positive				
No	203	179	24	
%	57.7%	88.2%	11.8%	1.000
**HER2/neu**				
Negative				
No	300	267	33	
%	87.7%	89.0%	11.0%	
Positive				
No	42	37	5	
%	12.3%	88.1%	11.9%	0.796
**Cmyc**				
Wild type				
No	407	353	54	
%	87.7%	86.7%	13.3%	
Amplified				
No	57	53	4	
%	12.3%	93.0%	7.0%	0.282
**GATA6**				
Wild type				
No	408	353	55	
%	89.7%	86.5%	13.5%	
Amplified				
No	47	42	5	
%	10.3%	89.4%	10.6%	0.819
**PIK3CA**				
Wild type				
No	398	351	47	
%	94.5%	88.2%	11.8%	
Amplified				
No	23	18	5	
%	5.5%	78.3%	21.7%	0.184
**Kras**				
Wild type				
No	388	335	53	
%	81.5%	86.3%	13.7%	
Amplified				
No	88	80	8	
%	18.5%	90.9%	9.1%	0.292

To analyze heterogeneity of ITGAV expression within the tumor we performed an analysis of 165 patients on our multi-spot TMA. A homogeneous distribution of ITGAV expression within the tumors was observed. The total frequency of ITGAV expression on the mulit-spot TMA was lower than on the single spot TMA. Eight on 165 patients were positive for ITGAV on at least 2 spots (4.8%), two of these patients showed no evidence of ITGAV expression at the infiltration zone There was a strong correlation between positive and negative patients on the single-spot and multi-spot TMA (p < 0.0001).

### ITGAV expression marks poor outcome in patients without neoadjuvant treatment

**Figure 2:**
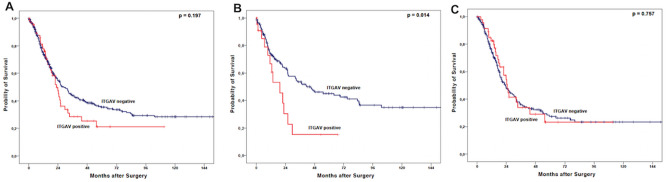
Kaplan–Meier survival analysis (log-rank test) for the entire patients’ cohort (**A**), patients after primary surgery (**B**) and patients after neoadjuvant treatment (**C**).

**Figure 3:**
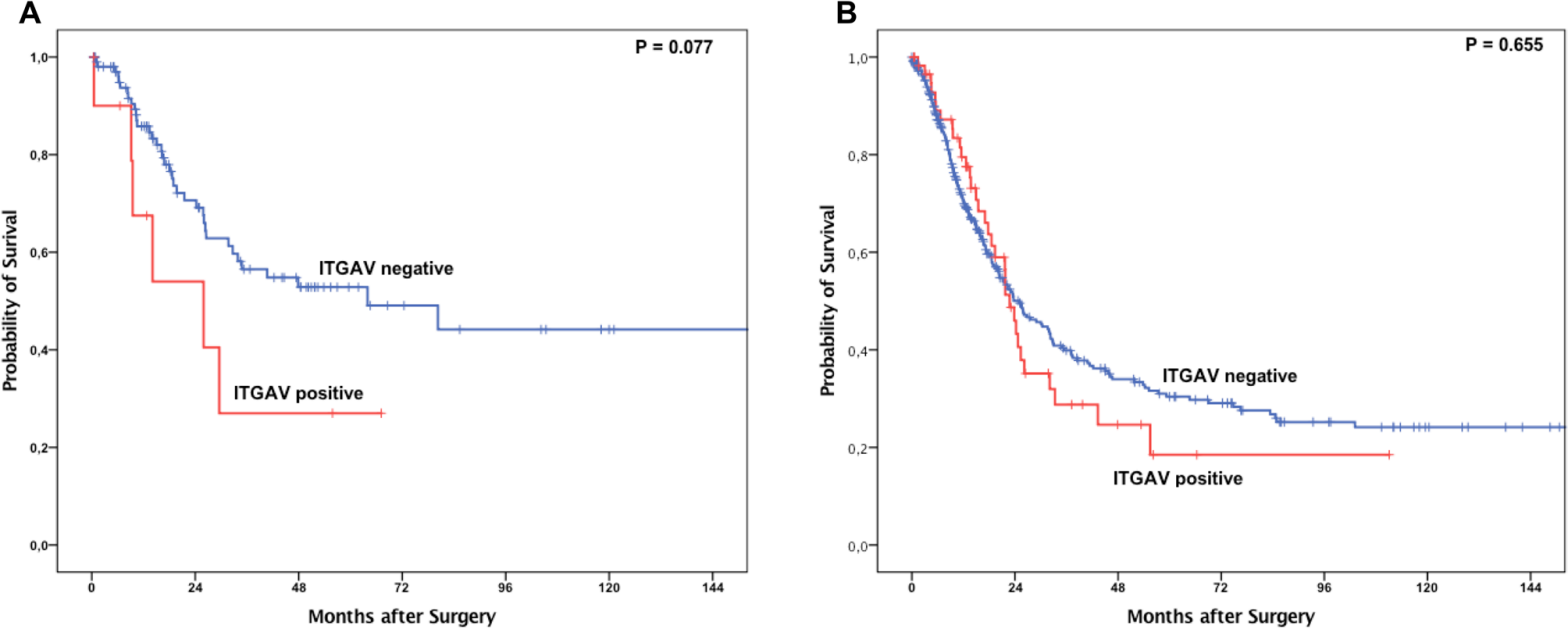
Kaplan–Meier survival analysis (log-rank test) for patients with pT1/2 tumors (**A**) and pT3/4 tumors (**B**).

Observing the entire patient cohort, a significant difference between patients with and without ITGAV expression could not be observed (Fig. 2A). However, in subgroup analysis, the group of patients that did not undergo neoadjuvant treatment, ITGAV expression was associated with a shortened overall-survival (OS). Patients without expression of ITGAV showed a median OS of 41.3 months (95% confidence interval (95% CI) 18.4–64.3 months) compared to a median OS of 19.3 months (95% CI 9.4–29.2 months, p = 0.014) in the group with ITGAV positive tumors. The effect was not seen in the group of patients after neoadjuvant treatment (p = 0.757) (Fig. 2B,C). Although there was no statistically significant difference, the survival analysis showed a distinct tendency towards a shorter survival in the group of pT1/2 carcinomas depending on ITGAV expression (Fig. 3A,B). An ITGAV depended difference in OS between patients with lymph node metastasis and those without could not be revealed (data not shown). To test whether the effects of ITGAV dependent survival differences are based on the correlation with tumor stages, a multivariate cox-regression model was performed which showed the tumor stage, lymph node metastasis and ITGAV expression as independent prognostic markers for overall-survival in the group of patients without neoadjuvant treatment (Table 3).

**Table 3:** Multivariate cox-regression model for patients after primary surgery and neoadjuvnat treatment.

	Primary surgery				Neoadjuvant treatment			
	Hazard ratio	95% confidence interval		p value	Hazard ratio	95% confidence interval		p value
		Lower	Upper			Lower	Upper	
Sex (male vs. female)	0.74	0.37	1.48	0.394	1.372	0.797	2.361	0.254
Age group (< 65yrs vs. > 65 yrs)	1.537	1.011	2.339	0.044	1.319	0.953	1.825	0.095
Tumor stage (pT1/2 vs. pT3/4)	2.057	1.208	3.504	0.008	0.922	0.593	1.433	0.718
Lymph node metastasis (pN0 vs. pN +)	3.641	2.228	5.951	< 0.001	2.571	1.771	3.732	< 0.001
Integrin alphaV expression (negative vs. positive)	2.031	1.102	3.741	0.023	0.951	0.618	1.464	0.819

## Discussion

**Figure 4:**
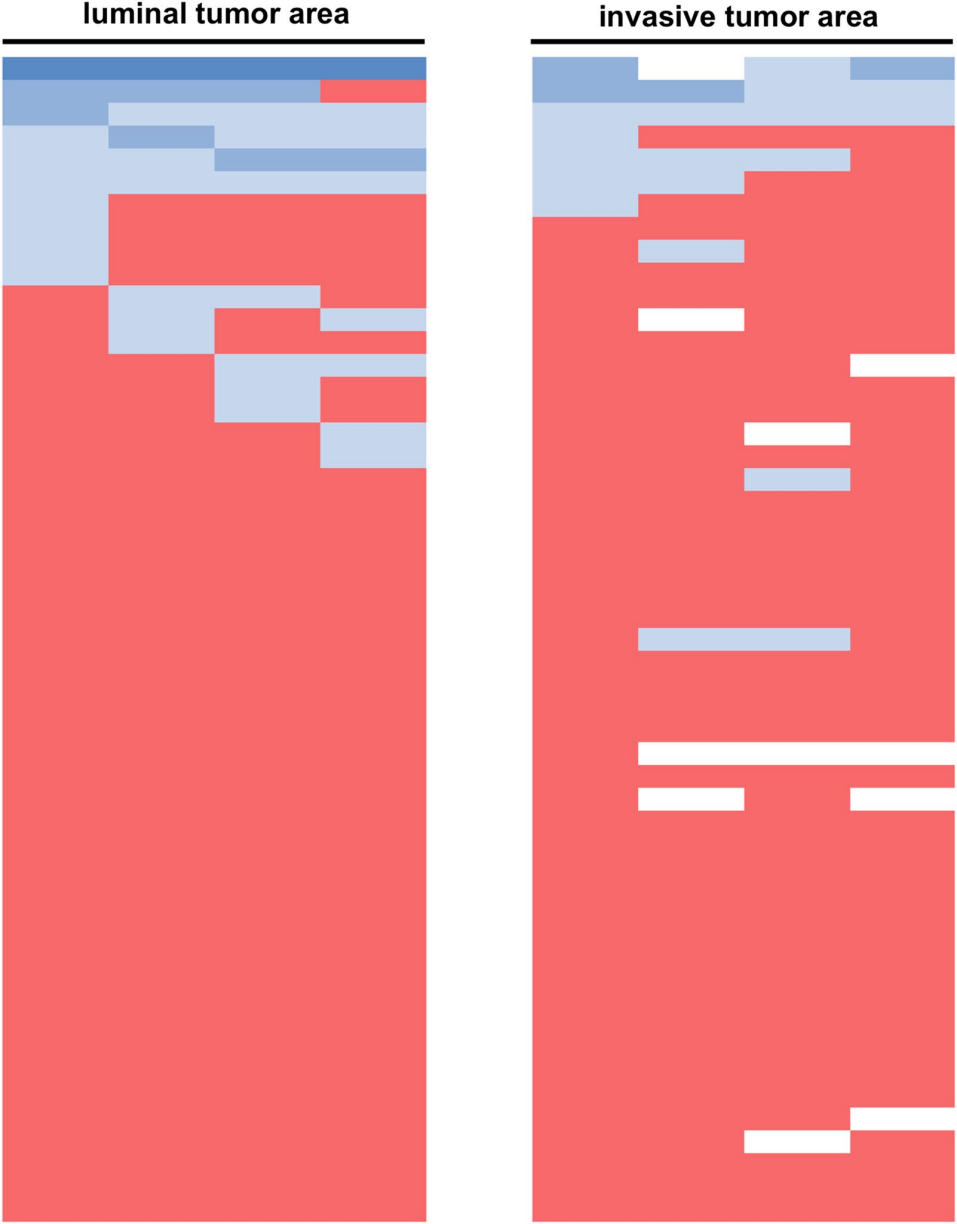
Visualization of the heterogeneity of ITGAV expression within the tumor by multi-spot TMA analysis. Each line represents an individual patient, each column a spot either on the luminal tumor area or the invasive tumor area. red = ITGAV negative, blue (light to dark) = weak to strong ITGAV expression.

EAC is one of the most aggressive gastrointestinal tumors and characterized by a high probability of metastasis as well as the occurrence of local recurrence after surgery. In the context of tumor progression, the interaction of tumor cells with the extracellular matrix is essential to ensure a tumor invasion into deeper tissue layers of the esophagus and therefore to establish a connection to the lymphatic and blood vessel system^22^. We can show in a large patient cohort of almost 600 EAC patients that the expression of ITAGV plays a role in tumor progression. It is associated with a poor OS in the group of patients without neoadjuvant therapy and serves as an independent prognostic marker (Fig. 4).

Integrins are natural candidates for analyzing the interaction between tumor cells and ECM because of their role as ligands for all major constitutes of the basement membrane and ECM^23^. The focus of this study lies in the analysis of the prognostic impact of ITGAV in the context with administration of neoadjuvant treatment in EAC patients. Under physiological circumstances, ITGAV is practically undetectable in normal tissue, however, it has been shown that ITGAV expression increases in a variety of epithelial tumors and its expression is associated with a poor prognosis^24^. ITGAV expression is found in about 16–18% of breast cancer^25,26^. Here it is remarkable that ITGAV is primarily detectable in advanced tumors, which underlines the potential role of integrin expression during tumor progression. In ovarian and pancreatic carcinoma nearly all tumors are positive for ITAGV expression, in colorectal carcinoma an expression of about 34–37% is found, in patients with synchronous liver metastasis in up to 70% of colorectal cancer patients^7,24,27,28^. So far, various mechanisms have been described to promote tumor progression by ITAGV. In particular, epithelial-mesenchymal transition (EMT), migration, cell proliferation and chemoresistance have been described as ITGAV mediated effects^29^. ITGAV could be identified as EMT marker in breast, colon and pancreatic carcinoma^30–32^. It was shown that the combination of integrin alpha V/beta 6 expression is closely correlated with the expression of other EMT markers, such as ZEB1 and ZEB2, and this could be explained as a possible pathway for ITAGV associated tumor cell detachment from rigid cell formation and therefore tumor progression in terms of metastatic spread^24^.

A further ITGAV meditated effect is that tumor cells with a high ITGAV expression show an increased cell migration in vivo and in vitro as well as an increased cell proliferation rate^9^. Furthermore, the cell invasion is increased by ITGAV mediated activation of matrix metalloproteinase 9 (MMP)^33^. The above-mentioned effects could have been primarily be attributed to an ITGAV-mediated TGF-beta activation^34,35^. TGF-b1 is a central mediator of tissue fibrosis and inflammation, and as an epithelial-restricted integrin, integrin αvβ6 expression is dramatically up-regulated in response to tissue injury and inflammation^36^. ITGAV-mediated TGF-beta activation is pivotal to the progression of various diseases such as fibrotic disease of the kidney and lung, pulmonary emphysema and acute lung injury^37^. TGF-beta-mediated tumor progression has already been demonstrated for various tumor entities. ITGAV-mediated TFG-beta activation is another activation pathway that has not been described for EAC.

Considering known molecular alterations in EAC we did not see any statistical correlation with TP53 mutation and Her2/neu, c-myc, GATA6, PIK3CA and KRAS amplification, although integrins are known to influence multiple signal pathways. For KRAS and PIK3CA, it is known that the expression of integrins can activate the RAS-MAPK-pathway and the PI3K-AKT-pathway, respectively^38,39^. In tumors that show an amplification of these genes (PIK3CA, KRAS GATA6) no statistically measurable additional effect can be achieved by ITAGV expression. For TP53 and Her2/neu, for example, there are contradictory data on the prognostic significance of these markers. At least in one operable tumor collective we and others could show that Her2/neu amplification of tumors is associated with a favorable prognosis^11,40^**.** According to the latest findings, TP53 mutations in EAC are associated with a so-called genomic catastrophe in at least part of the tumors, which is associated with chromotrypsis^41^ This mechanism probably affects up to one third of all EACs, so that interactions of TP53 and ITGAV that have been described in other tumor entities (see below) cannot necessarily be transferred to EACs. These remarks underline once again how multifactorial molecular changes are effective. For example, in colon cancer cells, the expression of Integrin5/beta1 mediates down-regulation of Her2/neu, suggesting a tumor suppressor function of αVβ6^6^.

It is known that mutant TP53 promotes recycling of Integrin and EGFR leading to activation of the AKT-pathway^42^. In colon cancer cells, the activation of TP53 inhibits expression of ITGAV, leading to cell survival^43^.

We can assume that especially considering KRAS, Her2/neu, GATA6 and PIK3CA the frequency of gene alterations in EAC is low (Table 2), and thus a probable influence of ITGAV cannot be shown statistically.

In this study we show that ITGAV expression is detectable in 14% of the EAC cases, which is somewhat lower than revealed in other tumor entities by TMA technique. Even though the TMA spots cover only small parts of the tumor, we have been able to show in the past that the expression in the TMA spot, especially in large number of patients, matches very well with the expression of the total tumor. We could show that there is a high concordance between the expression in the single-spot TMA for ITGAV expression and the expression on the multi-spot TMA and therefore we conclude that the TMA technique is appropriate to assess ITGAV expression pattern in esophageal adenocarcinoma.

Neoadjuvant chemo-(radio) therapy concepts have no influence on the expression frequency, no significant difference in the number of positive or negative tumor samples was detectable. Interestingly, there is no ITGAV associated influence on overall survival in the overall cohort, but there is a significant survival difference in the group of patients with primary surgery. Here a clear survival disadvantage is found if ITGAV is present in the tumor cells. This effect is explainable as a driver of tumor progression, EMT and signaling as described above. This clearly identifies ITGAV as a promoter of tumor progression for EAC. Currently, we can only speculate why the survival difference is not detectable in the group of patients after neoadjuvant therapy. It is possible that neoadjuvant therapies promote fundamental structural epigenetic changes of tumor cells, so that the ITAGV meditated effects cannot be detected well by immunohistochemical analysis^44^. Of course, due to the retrospective nature of the study the influence of perioperative therapy on ITGAV-associated effects can only be explained indirectly. Further prospective analyses are necessary, for example to investigate the significance of ITGAV expression on biopsy material. However, the effects in the group of primarily operated patients are clear and show a significantly shortened overall survival in cases with ITGAV expression, so that ITGAV may play a role as a prognostic tumor marker in the description of disease progression of EAC.

## Data Availability

Data available on request due to privacy/ethical restrictions.
